# Efficacy of Resveratrol Supplementation against Non-Alcoholic Fatty Liver Disease: A Meta-Analysis of Placebo-Controlled Clinical Trials

**DOI:** 10.1371/journal.pone.0161792

**Published:** 2016-08-25

**Authors:** Chongyang Zhang, Weigang Yuan, Jianguo Fang, Wenqing Wang, Pei He, Jiahui Lei, Chunxu Wang

**Affiliations:** 1 Department of Pathogenic Biology, School of Basic Medicine, Tongji Medical College, Huazhong University of Science and Technology, Wuhan, Hubei, China; 2 Department of Anthropotomy, School of Basic Medicine, Tongji Medical College, Huazhong University of Science and Technology, Wuhan, Hubei, China; 3 Department of Pharmacy, Tongji Hospital Affiliated with Tongji Medical College, Huazhong University of Science and Technology, Wuhan, Hubei, China; 4 Department of Obstetrics and Gynecology, Wuhan NO.1 Hospital Affiliated with Tongji Medical College, Huazhong University of Science and Technology, Wuhan, Hubei, China; University of Catania, ITALY

## Abstract

Non-alcoholic fatty liver disease (NAFLD) is the most common chronic liver disease with rising prevalence. Increasing evidence has demonstrated that resveratrol, a dietary phytochemical, is capable of attenuating NAFLD development and progression; however, results from clinical studies are inconsistent and inconclusive. Here, we conducted a meta-analysis to evaluate the efficacy of resveratrol on NAFLD, using several parameters to provide new insights for clinical application. We systematically searched EMBASE, PubMed, Science Citation Index, Elsevier, and Cochrane Library databases for studies published up to date (July 2016), in English, to identify and screen eligible, relevant studies. Either a fixed-effect model or random model was used to estimate mean difference (MD) and 95% confidence intervals (CIs) for the effect of resveratrol on NAFLD. Four randomized, double-blinded, placebo-controlled trials involving 156 patients were included in the meta-analysis. Levels of low-density lipoprotein (MD = 0.47, 95% CI: 0.21, 0.74, *P* < 0.05) and total cholesterol (MD = 0.49, 95% CI: 0.18, 0.80, *P* < 0.05) were higher in the resveratrol treatment groups than in placebo control groups, whereas other parameters were not altered. Overall, this study indicates that resveratrol treatment has negligible effects on attenuating NAFLD, given the small improvement in NAFLD features. More high-quality clinical trials of resveratrol for NAFLD are required to confirm these results.

## Introduction

Nonalcoholic fatty liver disease (NAFLD), a spectrum of liver injuries ranging from simple steatosis to nonalcoholic steatohepatitis (NASH), is now the most prevalent chronic liver disease in the overweight population, affecting about one-third of the population in the Western world and Asia [1, 2]. Increasing body weight and obesity have been identified as key risk factors for many metabolic diseases, including cardiovascular diseases, type 2 diabetes and lipid disorders, and NAFLD [3]. More importantly, NAFLD is strongly associated with abdominal obesity, insulin resistance (IR), excessive inflammation, dyslipidemia, arterial hypertension, and chronic kidney disease [4, 5]. Universally accepted therapeutic options other than lifestyle modifications, including weight reduction diets and exercise, are not yet available [6]. Therefore, an effective treatment method for NAFLD is needed to overcome these limitations [7, 8].

Recently, studies have suggested that some natural products may have antioxidant effects on steatohepatitis [9,10]; however, the specific effects of resveratrol on NAFLD are controversial. Resveratrol (3,5,4′-trihydroxy-trans-stilbene) is a natural polyphenol found in grapes and red wine that has been shown to extend lifespan in many organisms [11, 12]. In the last few years, resveratrol has been characterized as having cardioprotective [13, 14], anti-inflammatory [15], and antioxidant[16] properties. *In vitro* and *in vivo* studies have suggested that resveratrol protects against metabolic phenotypes by activating silent information regulation 2 homologue 1 (SIRT1) [17] and AMP activated protein kinase (AMPK) [18], thereby mimicking a condition of caloric restriction (CR) [19]. This raises the possibility for the use of resveratrol as an agent to treat obesity and obesity-associated diseases such as NAFLD. It is reported that resveratrol has promising inhibitory effects on the development of NAFLD by functioning against lipid accumulation induced by a high-fat diet [20]. However, two recent double-blinded, randomized, and placebo-controlled clinical trials (RCTs) showed that resveratrol treatment has no consistent therapeutic efficacy for alleviating clinical or histological NAFLD [21, 22].

Although resveratrol has numerous beneficial effects on humans, whether it has beneficial effects on patients with NAFLD is unknown, and recent studies have shown paradoxical results on the efficacy of resveratrol for treating NAFLD. Therefore, we conducted this comprehensive meta-analysis of qualified RCTs to investigate whether resveratrol supplementation is beneficial for patients with NAFLD. The study design was formulated by face-to-face discussions with all authors until a consensus was reached. We believe that this study will provide new insights into the use of resveratrol in NAFLD and function as a foundation to expedite development of treatment for NAFLD.

## Methods

We performed this meta-analysis according to the Preferred Reporting Items for Systematic Reviews and Meta-analyses (PRISMA) guidelines [23]; a detailed protocol was established before beginning the study. The study objectives were met by ensuring that all studies included in this analysis involved NAFLD patients or healthy people (participants) with or without resveratrol treatment (intervention) from randomized controlled clinical studies. We evaluated the efficacy of resveratrol supplementation against NAFLD by comparing effects of resveratrol at any dosage with those of placebo or no intervention; the outcomes were pooled by using either a fixed-effect model or random model to estimate mean difference (MD) and 95% confidence intervals (CIs).

### Search strategies

A computerized search of EMBASE (www.embase.com), PubMed (http://www.ncbi.nlm.nih.gov/pubmed), Science Citation Index (http://thomsonreuters.com/en/products-services/scholarly-scientific-research/scholarly-search-and-discovery/science-citation-index-expanded.html), Elsevier (http://www.sciencedirect.com/), and the Cochrane Library (www.cochranelibrary.com) was conducted for English-language studies published up to July 2016. The literature search was performed using combinations of database-specific search terms for NAFLD (Nonalcoholic Fatty Liver Disease or NAFLD or Nonalcoholic Fatty Liver Disease or Fatty Liver, Nonalcoholic or Fatty Livers, Nonalcoholic or Liver, Nonalcoholic Fatty or Livers, Nonalcoholic Fatty or Nonalcoholic Fatty Liver or Fatty Liver, Nonalcoholic or Fatty Livers, Nonalcoholic or Liver, Nonalcoholic Fatty or Livers, Nonalcoholic Fatty or Nonalcoholic Fatty Liver or Nonalcoholic Fatty Livers or Nonalcoholic Steatohepatitis or Steatohepatitides, Nonalcoholic or Steatohepatitis, Nonalcoholic) and resveratrol or resveratrols. To be eligible for inclusion in this study, the keywords were required to appear in the title or/and abstract upon retrieval. The search was restricted to studies in human RCTs investigating the potential efficacy of resveratrol supplementation on NAFLD. Unpublished data were obtained by contacting authors of relevant abstracts (if available). All reference sections of qualified studies and relevant reviews were hand-reviewed for potential studies. Two authors (C-Y.Z. and W-G.Y.) assessed each manuscript. Consensuses were achieved by agreement and discussion with a third reviewer (J-H.L.).

### Study selection

Two authors (C-Y.Z. and W-G.Y.) independently reviewed the titles and abstracts of original articles to screen eligible studies that met the inclusion criteria. In case of any discordance, a third reviewer (C-X.W.) was consulted. Studies that met the following criteria were included: (1) published research articles with completed RCTs reported by original articles or meeting articles (article type); (2) individuals suffering from NAFLD (participants); and (3) studies comparing the effects of resveratrol at any dosage with those of placebo or no intervention (intervention and comparators).

The following studies were excluded: (1) repeated reports with the same first author; (2) reports missing complete outcome data; (3) reports that did not qualify as a RCT; and (4) reports in which the data could not be extracted by current mathematical methods.

### Data extraction

Two authors (C-Y.Z. and W-G.Y.) independently extracted study data by using a standardized data collection form that included the full name of the first author, publication date, study design, origin country, sample size, age, race/ethnicity, outcome assessment, weighted- and/or standardized-mean difference (MD) and the corresponding 95% CIs, response rates (RRs), statistical adjustment for the major confounding factors, statistical methods applied for the analysis, and relevant parameters of NAFLD. The control groups from all studies included in this analysis were treated with placebo or no intervention. The extracted data were verified by a third author (C-X.W.); disagreements were resolved by face-to-face discussion between the three authors. The same reviewers were contacted by telephone or email when questions arose regarding their articles.

### Quality assessment

The Cochrane Risk of Bias assessment tool was independently performed by two authors (C-Y.Z. and W-G.Y.) to assess the quality of the studies. The items included random sequence generation, allocation concealment, blinding of participants and personnel, blinding of outcome assessment, incomplete outcome data, selective reporting, and other bias. Disagreements were adjudicated by discussion until an agreement was reached.

### Statistical analysis

All statistical analyses were performed using the Review Manager 5.3 software. We computed the MD and 95% CIs from the estimates reported in each eligible study. Heterogeneity was quantified by the Cochran’s Q and the *I*^*2*^ statistics (I^2^ > 50% was used as a threshold indicating significant heterogeneity). Publication bias was assessed with Funnel plots and Egger’s regression model. Data from the studies were combined using a fixed-effects model or a random-effects model. The Mantel and Haenszel method in fixed-effects model and DerSimonian and Laird method in random-effect model were applied to obtain a pooled estimate of the effect size [24, 25]. Pooled *P* values < 0.05 were considered significant. We performed sensitivity analysis owing to the considerable heterogeneity. After omitting Faghihzadeh *et al*.’s study [26] on systolic blood pressure (SBP) and low-density lipoprotein (LDL) levels and Heebøll *et al*.’s study [22] on diastolic blood pressure (DBP), the heterogeneity was acceptable.

### Study quality and publication bias

Study quality was independently evaluated by two reviewers (C-Y.Z. and W-G.Y.). First, we carefully reviewed the four included studies to determine study quality. We next performed a detailed checklist of items, using the Cochrane Risk of Bias assessment tool. The population was described as any adult with NAFLD. Potential confounders including age, gender, baseline biochemistry, change in diet, and additional exercise were also considered. When reviewers disagreed, a consensus was reached by discussing with a third reviewer (C-X.W.).

The funnel plots were symmetrical and Egger’s test results showed no significant publication bias in the current meta-analysis (Egger’s test: *P* > 0.05) (S1 Fig).

### Sensitivity analysis

Results of sensitivity analysis demonstrated that the significant estimate of pooled RRs was not significantly affected by omitting every single study each time (data not shown).

## Results

### Study selection

**Fig 1:**
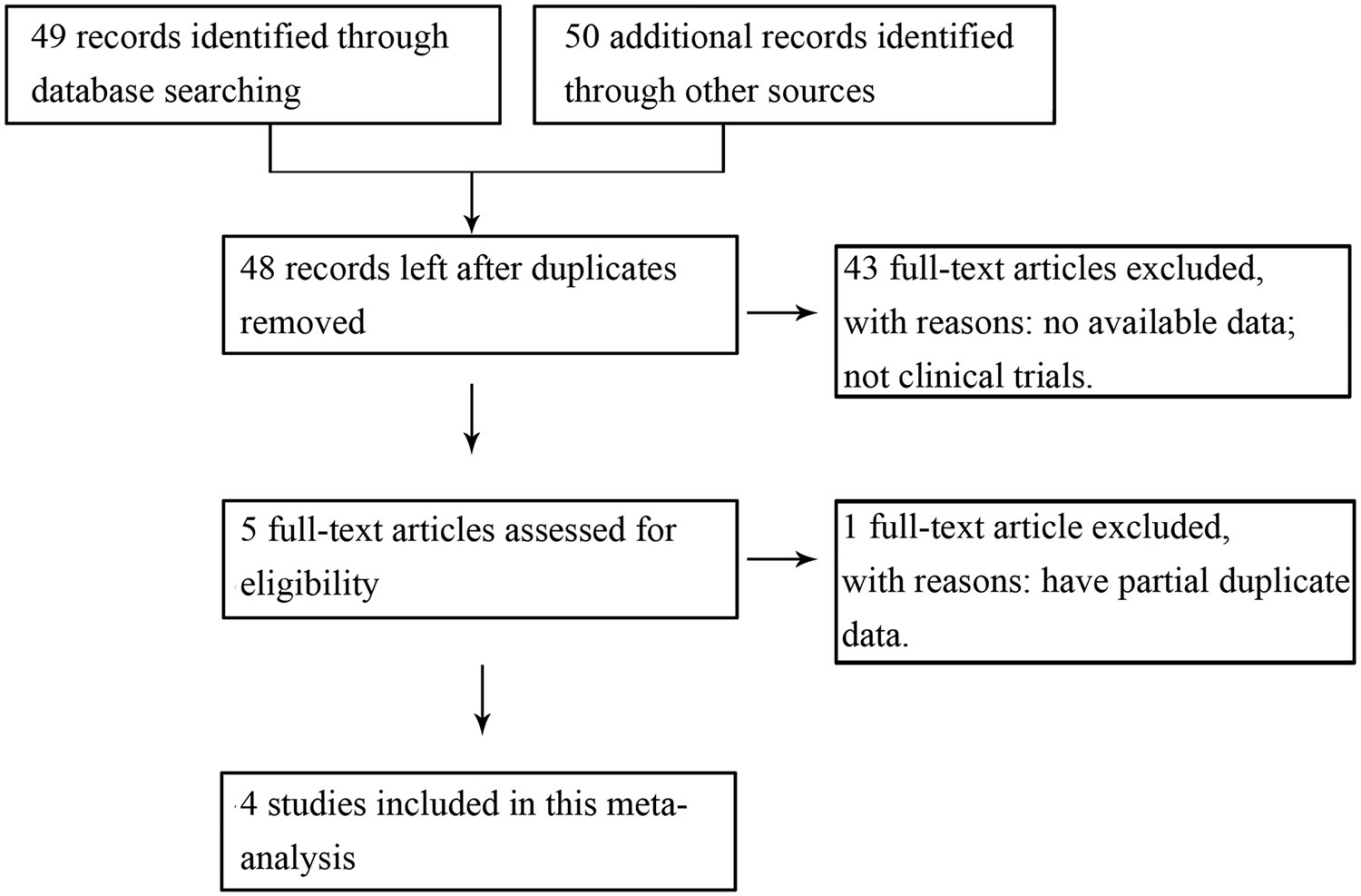
Flowchart of study inclusions and exclusions.

We initially retrieved 49 records identified through database searching and identified 50 additional records through other sources. After removing duplicates, 48 studies were potentially relevant. After a conscientious review of the abstract or full-text, 43 full studies were excluded because they were not clinical trials or had no available data. The remaining five studies were considered potentially eligible for the meta-analysis. Among them, two studies reported by Faghihzadeh *et al*. had partial duplicate data; consequently, the study published in 2015 was selected owing to its relatively comprehensive data [26, 27]. Thus, four studies published from 2014 to 2016 were included in the present meta-analysis (Fig 1).

### Characteristics of the studies included

Table 1 presents the characteristics of participants in the eligible studies. We identified a total of 156 participants from the four included studies. The range of enrollment periods for participants across the included studies was from October 2011 to September 2014. All the patients had NAFLD. Indications of publication bias were not statistically significant. The PRISMA checklist is shown in S1 Table.

**Table 1:** Characteristics of patients in the included studies.

Author, year [reference]	Therapy	Usage	Age	N	Time (day)
Faghihzadeh et al. 2015 [26]	Resveratrol group	a 500-mg resveratrol capsule	44.04±10.10	25 (7 females)	84
	Placebo group	a placebo capsule	46.28±9.52	25 (8 females)	
Heebøll et al. 2016 [22]	Resveratrol group	500 mg, three times daily	no data	13	180
	Placebo group	Placebo	no data	13	
Chen et al. 2015 [7]	Resveratrol group	2 150 mg resveratrol capsules	45.2±10	30 (8 females)	90
	Placebo group	2 placebo capsules	43.5±11.0	30 (10 females)	
Chachay et al. 2014 [21]	Resveratrol group	3000 mg resveratrol daily	48.8±12.2	10	56
	Placebo group	Placebo	47.5±11.2	10	

### Effects of resveratrol on NAFLD

#### Anthropometric index and clinical parameters

**Fig 2:**
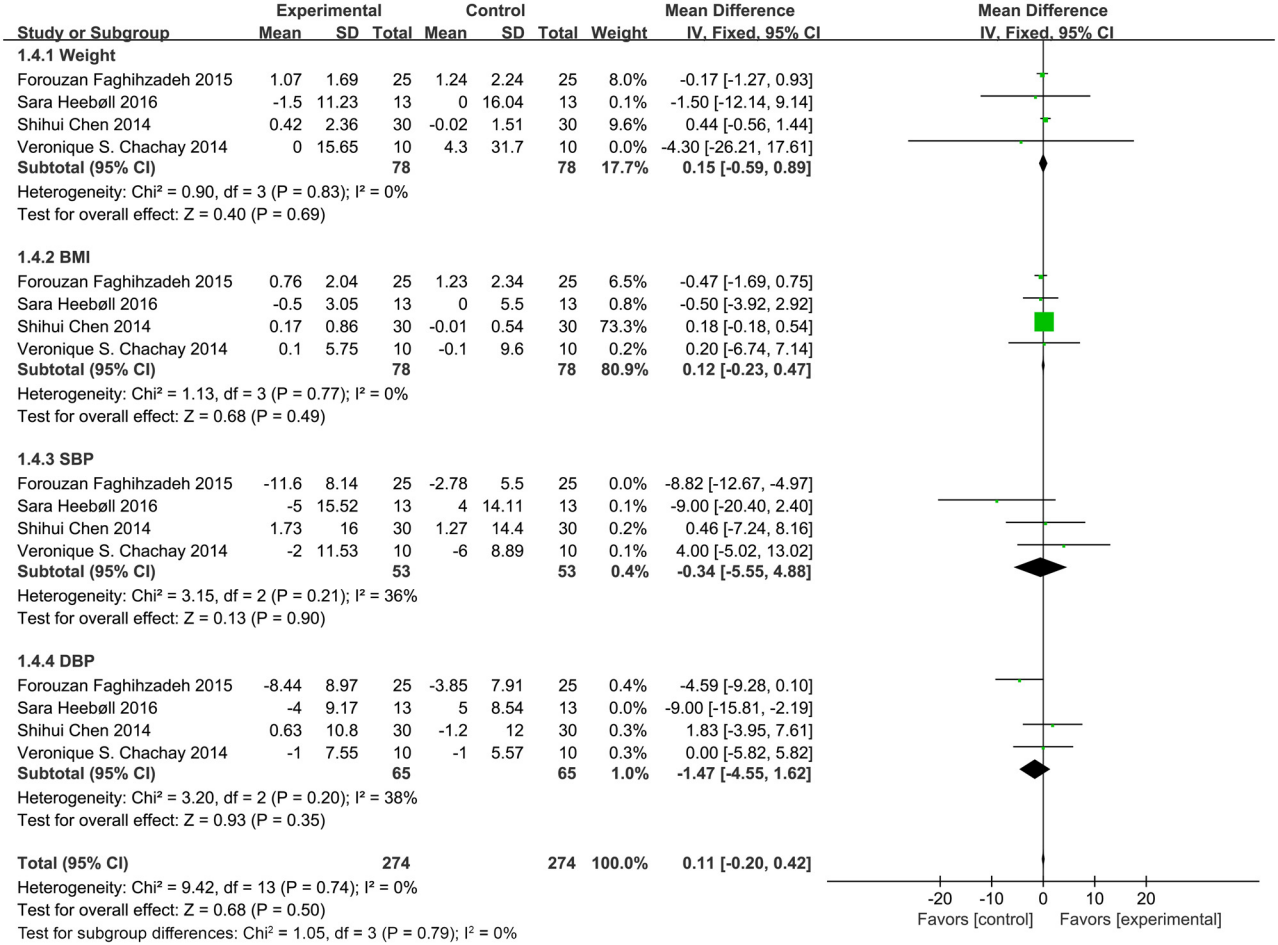
Meta-analysis with studies reporting effect of resveratrol supplementation on anthropometric index (BMI, body mass index) and clinical parameters (SBP, systolic blood pressure; DBP, diastolic blood pressure) using a fixed-effects model. CI indicates confidence interval.

Four parameters—weight, body mass index (BMI), DBP, and SBP—were utilized to evaluate the anthropometric index and clinical parameters. Each parameter included four trials. Compared with control groups, no significant differences were observed for weight (MD = 0.15, 95% CI: -0.59, 0.89, *P* = 0.69), BMI (MD = 0.12, 95% CI: -0.23, 0.47, *P* = 0.49), DBP (MD = -1.47, 95% CI: -4.55, 1.62, *P* = 0.35), and SBP (MD = -0.34, 95% CI: -5.55, 4.88, *P* = 0.90) levels after resveratrol treatment (Fig 2).

#### Lipid metabolic parameters and glucose metabolic parameters

**Fig 3:**
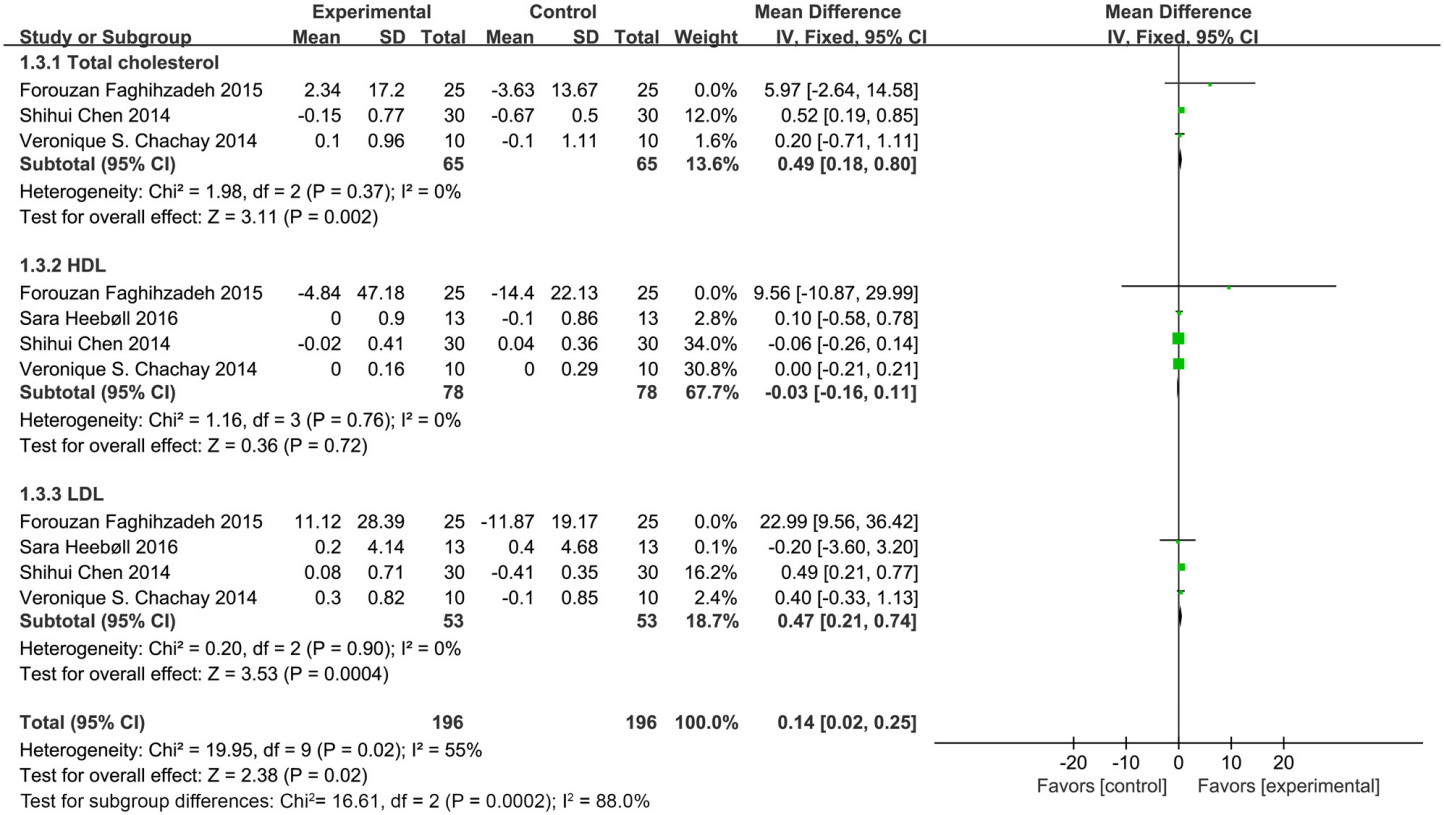
Meta-analysis of studies reporting an effect of resveratrol supplementation on lipid metabolic parameters (total cholesterol; HDL, high-density lipoprotein; LDL, low-density lipoprotein) using a fixed-effects model. CI indicates confidence interval.

**Fig 4:**
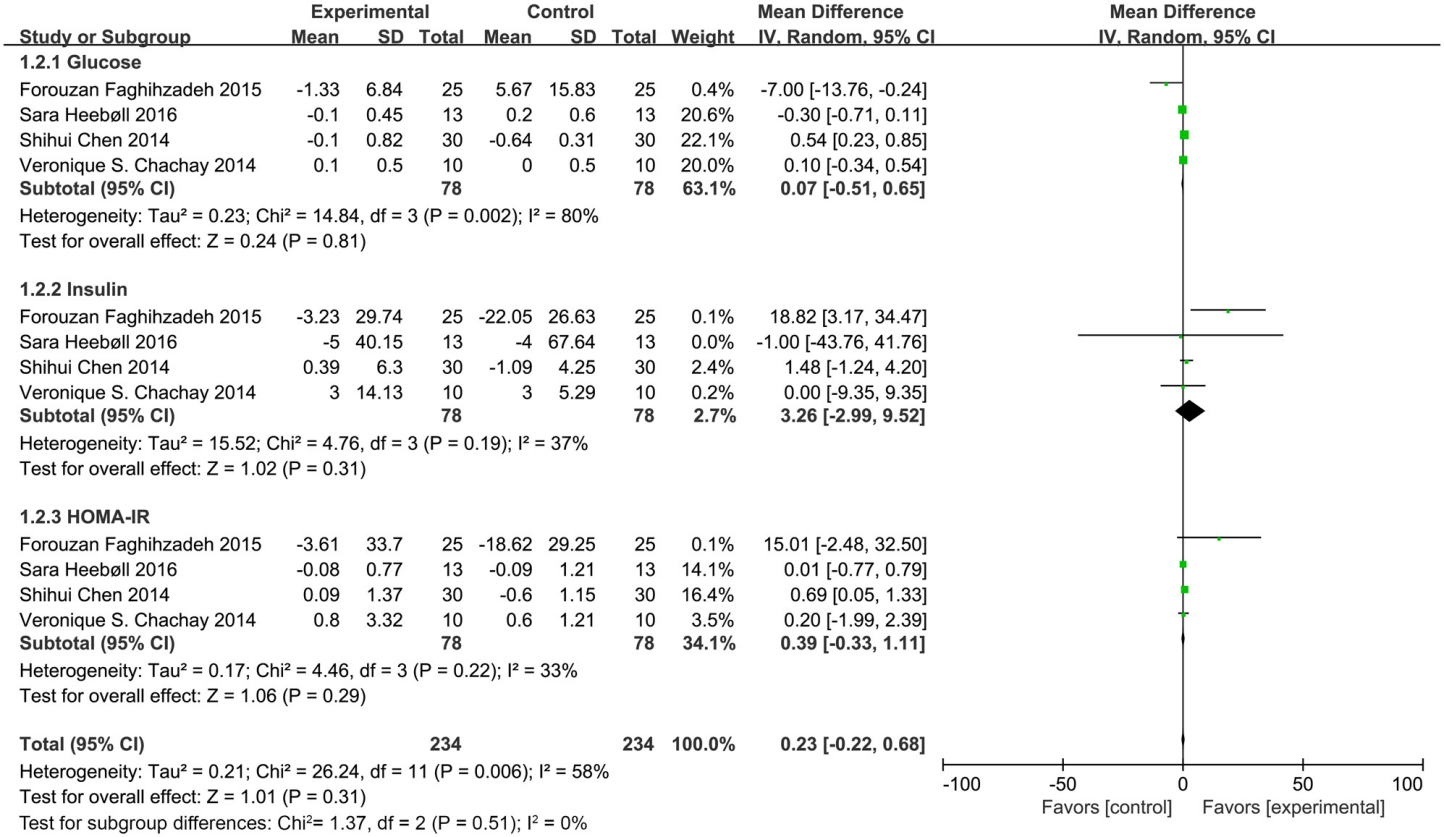
Meta-analysis of studies reporting an effect of resveratrol supplementation on glucose metabolic parameters (glucose; insulin; HOMA-IR, homeostatic model assessment of insulin resistance) using a random-effects model. CI indicates confidence interval.

As shown in Fig 3, total cholesterol (MD = 0.49, 95% CI: 0.18, 0.80, *P* < 0.05) and LDL (MD = 0.47, 95% CI: 0.21, 0.74, *P* < 0.05) levels were significantly higher in subjects supplemented with resveratrol than control subjects. We excluded a study of Faghihzadeh *et al*. [26] owing to the considerable heterogeneity presented in LDL levels. The heterogeneity test yielded a value of *I*^*2*^ = 0% and *P* = 0.90 in Cochran's test as well; however, the LDL levels still had statistical significance. The mean difference in lipid metabolic parameters and glucose metabolic parameters was reported in four trials, with total cholesterol reported in three trials. In addition, no significant differences were detected in levels of high-density lipoprotein (HDL) (MD = -0.03, 95% CI: -0.16, 0.11, *P* = 0.72), glucose (MD = 0.07, 95% CI: -0.51, 0.65, *P* = 0.81), insulin (MD = 3.26, 95% CI: -2.99, 9.52, *P* = 0.31), and homeostatic model assessment of insulin resistance (HOMA-IR) (MD = 0.39, 95% CI: -0.33, 1.11, *P* = 0.29) (Fig 4). We used the random model to assess glucose metabolic parameters owing to the significant heterogeneity.

#### Liver enzymes

**Fig 5:**
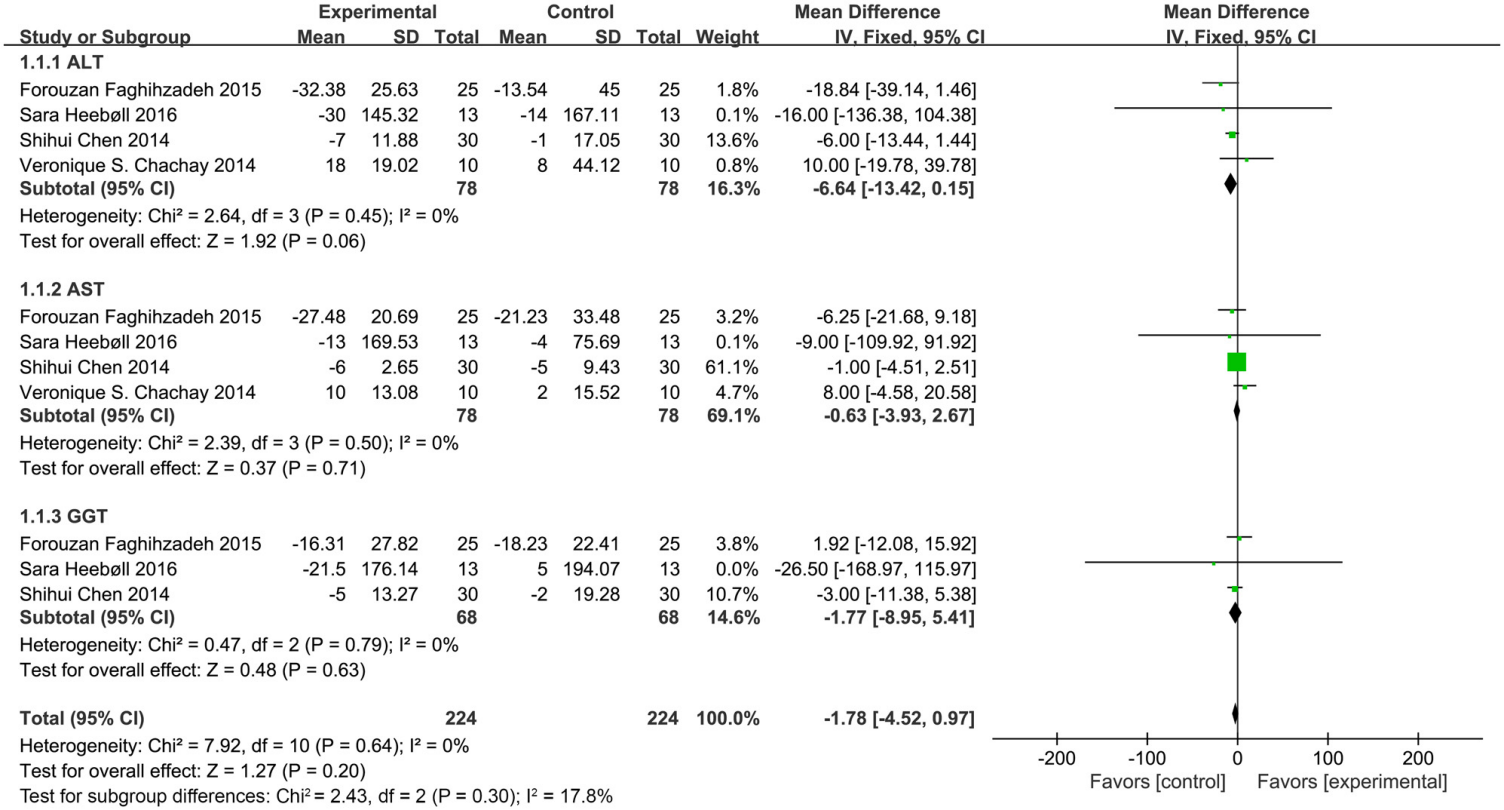
Meta-analysis of studies reporting an effect of resveratrol supplementation on levels of liver enzymes (ALT, alanine aminotransferase; AST, aspartate aminotransferase; GTT, γ-glutamyltransferase) using a fixed-effects model. CI indicates confidence interval.

Three trials reported γ-glutamyltransferase (GGT) levels and four reported serum aspartate aminotransferase (AST) and alanine aminotransferase (ALT) levels. As revealed in Fig 5, none of the three parameters, including GGT (MD = -1.77, 95% CI: -8.95, 5.41, *P* = 0.63), AST (MD = -0.63, 95% CI: -3.93, 2.67, *P* = 0.71), and ALT (MD = -6.64, 95% CI: -13.42, 0.15, *P* = 0.06) showed statistically significant differences among the control and resveratrol-treated groups.

#### Bilirubin and tumor necrosis factor α (TNF-α)

**Fig 6:**
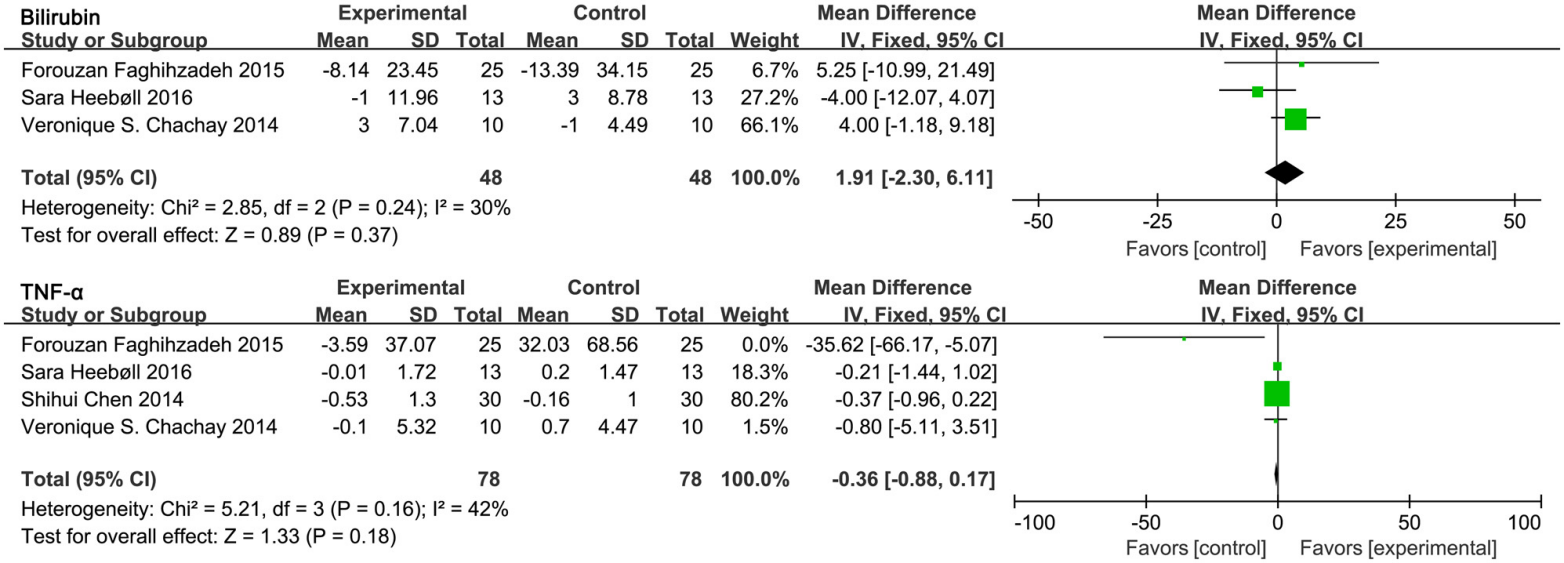
Meta-analysis of studies reporting an effect of resveratrol supplementation on bilirubin and TNF-α (tumor necrosis factor α) levels using a fixed-effects model. The upper section indicates bilirubin levels and the lower section reveals TNF-α levels. CI indicates confidence interval.

Three out of four RCTs reported data on bilirubin levels and four reported TNF-α levels. Fig 6 shows that both bilirubin (MD = 1.91, 95% CI: -2.30, 6.11, *P* = 0.37) and TNF-α (MD = -0.36, 95% CI: -0.88, 0.17, *P* = 0.18) levels were not significantly different between the control and resveratrol-treated groups.

### Risk of bias across studies

We evaluated the risk of bias and random errors by applying the following seven considerations: random sequence generation, allocation concealment, blinding of participants and personnel, blinding of outcome assessment, incomplete outcome data, selective reporting, and other bias. Risk of bias and random errors summary and graph, as well as reviewers' judgments on each risk-of-bias item for each included study are presented in S2 and S3 Figs.

## Discussion

To date, there is no proven medical therapy for NAFLD [28]. In spite of the growing number of interpretations of the pathogenic mechanism of NAFLD, effective therapeutic agents are still lacking. It is therefore of great urgency to find suitable treatment measures [29, 30]. Resveratrol is a polyphenol found in a variety of plant species. An accumulating number of high-quality preclinical studies have confirmed its efficacy for reducing hepatic lipogenesis, resulting in inactivation of liver X receptor α (LXRα) and inhibition of TNF-α, thereby preventing hepatic steatosis [20, 31]. Nonetheless, clinical trials regarding the efficacy of resveratrol supplementation in NAFLD have shown conflicting results. In previous RCTs, Chachay *et al*. demonstrated that resveratrol treatment significantly increased the levels of AST and ALT, but did not improve any features of NAFLD [21]. Faghihzadeh *et al*. showed that resveratrol supplementation did not have any beneficial effect on anthropometric measurements, lipid profile and blood pressure, or insulin resistance, but reduced ALT levels and hepatic steatosis in patients with NAFLD [26]. Heebøll *et al*. revealed that resveratrol treatment had no consistent therapeutic effect in relieving clinical or histological NAFLD, but displayed a negligible ameliorating effect on liver functions and liver fat accumulation [22]. Chen *et al*. showed that resveratrol significantly decreased the levels of TNF-α, LDL, ALT, total cholesterol, and HOMA-IR [7]. Given these controversial results, this meta-analysis was conducted to provide reliable evidence for the efficacy of resveratrol supplementation in NAFLD. Although the present study did find some positive effects of resveratrol on metabolic parameters, the improvement in liver function and fatty liver was less apparent than expected.

In this study, glucose metabolism parameters (glucose, insulin, and HOMA-IR) were unchanged after resveratrol treatment, which is consistent with other studies [21, 22, 27]. For the lipid metabolic parameters (total cholesterol, LDL, and HDL), our results showed that resveratrol supplementation increased total cholesterol and LDL levels, which is noteworthy and in contrast to the study reported by Chen *et al*. [7].

It has been documented that the inflammatory mediator TNF-α plays a vital role in the pathogenesis of NAFLD. Previous studies have shown that elevated TNF-α levels are an independent predictor of histological fibrosis in patients with NAFLD. Our results show that supplementation with resveratrol has no effect on TNF-α levels. This result is in contrast with that of Chen *et al*. [7]. In addition, bilirubin levels were not changed significantly following resveratrol treatment, which is in conflict with the result of Faghihzadeh *et al*. [26].

For the anthropometric index and clinical parameters, we evaluated weight, BMI, SBP, and DBP and found that none of them were changed after resveratrol treatment. These findings are in accordance with those of some previous studies [7, 21, 27]. Timmers *et al*. reported that 30 days of 150 mg/d resveratrol supplementation reduced SBP levels, and Bhatt *et al*. showed that 3 months of 250 mg/g resveratrol supplementation had the same effect on patients with type 2 diabetes [32, 33]. However, the included four individual studies in our meta-analysis showed no significant changes in SBP levels. These paradoxical results may be attributed to the different dose of resveratrol administered in the different studies. More high-quality studies are therefore required to determine the dose-dependent effects of resveratrol.

For liver enzymes (AST, ALT, and GGT), this meta-analysis showed that resveratrol had no effects on liver enzyme activity. Heebøll *et al*. consistently demonstrated that resveratrol treatment does not influence ALT levels [22]. However, Faghihzadeh *et al*. showed that 12 weeks of supplementation with 500 mg of resveratrol reduces ALT levels [26], and Chen *et al*. suggested that resveratrol significantly decreased AST and ALT levels [7]. On the contrary, Chachay *et al*. revealed that AST and ALT levels were elevated after resveratrol treatment for 6 weeks [21]. These previous results are controversial. Thus, larger-scale, well-controlled studies are urgently needed to confirm these results.

The present study has a few strengths and limitations. This meta-analysis only includes RCTs and no time limitations were considered for the literature search. There is no risk of bias and random errors in this meta-analysis and the parameters we evaluated are relatively comprehensive. However, the sample size was relatively small, which may have biased the results to some extent. In addition, the differences in dosages of resveratrol and duration of the studies may also have affected the accuracy of the results.

In conclusion, this study provides novel insights into the beneficial effects of resveratrol supplementation in patients with NAFLD. It reveals that resveratrol significantly increases LDL and total cholesterol levels, providing evidence to support that the drug may ameliorate lipid metabolic parameters. However, resveratrol showed no beneficial influence on other parameters. To better understand the efficacy of resveratrol in patients with NAFLD, large-scale, well-designed, and population-based studies are required in the future.

## Supporting Information

S1 Fig Funnel plots displaying the anthropometric index and clinical parameters, lipid metabolic parameters, glucose metabolic parameters, liver enzymes, TNF-α, and bilirubin. (TIF)

S2 Fig Risk of bias and random errors summary: reviewers' judgments on each risk-of-bias item across all included studies. (TIF)

S3 Fig Risk of bias and random errors graph: reviewers' judgments on each risk-of-bias item across all included studies. (TIF)

S1 Table The PRISMA statement for reporting systematic reviews and meta-analyses of studies. (DOCX)

## References

[1] Loomba R, Sanyal AJ. The global NAFLD epidemic. Nat Rev Gastroenterol Hepatol. 2013;10: 686–690. 10.1038/nrgastro.2013.171 24042449

[2] Vernon G, Baranova A, Younossi ZM. Systematic review: the epidemiology and natural history of non-alcoholic fatty liver disease and non-alcoholic steatohepatitis in adults. Aliment Pharmacol Ther. 2011;34: 274–285. 10.1111/j.1365-2036.2011.04724.x 21623852

[3] Marchesini G, Babini M. Nonalcoholic fatty liver disease and the metabolic syndrome. Minerva Cardioangiol. 2006;54: 229–239. 16778754

[4] Tilg H, Moschen AR. Evolution of inflammation in nonalcoholic fatty liver disease: the multiple parallel hits hypothesis. Hepatology. 2010;52: 1836–1846. 10.1002/hep.24001 21038418

[5] Lonardo A, Sookoian S, Chonchol M, Loria P, Targher G. Cardiovascular and systemic risk in nonalcoholic fatty liver disease—atherosclerosis as a major player in the natural course of NAFLD. Curr Pharm Des. 2013;19: 5177–5192. 23432668

[6] Dudekula A, Rachakonda V, Shaik B, Behari J. Weight loss in nonalcoholic Fatty liver disease patients in an ambulatory care setting is largely unsuccessful but correlates with frequency of clinic visits. PloS One. 2014;9: e111808 10.1371/journal.pone.0111808 25375228 PMC4222918

[7] Chen S, Zhao X, Ran L, Wan J, Wang X, Qin Y, et al Resveratrol improves insulin resistance, glucose and lipid metabolism in patients with non-alcoholic fatty liver disease: A randomized controlled trial. Dig Liver Dis. 2015;47: 226–232. 10.1016/j.dld.2014.11.015 25577300

[8] Nagao K, Inoue N, Inafuku M, Shirouchi B, Morooka T, Nomura S, et al Mukitake mushroom (*Panellus serotinus*) alleviates nonalcoholic fatty liver disease through the suppression of monocyte chemoattractant protein 1 production in db/db mice. J Nutr Biochem. 2010;21: 418–423. 10.1016/j.jnutbio.2009.01.021 19423319

[9] Salamone F, Galvano F, Marino Gammazza A, Paternostro C, Tibullo D, Bucchieri F, et al Silibinin improves hepatic and myocardial injury in mice with nonalcoholic steatohepatitis. Dig Liver Dis. 2012;44: 334–342. 10.1016/j.dld.2011.11.010 22197629

[10] Salamone F, Galvano F, Cappello F, Mangiameli A, Barbagallo I, Li Volti G. Silibinin modulates lipid homeostasis and inhibits nuclear factor kappa B activation in experimental nonalcoholic steatohepatitis. Transl Res. 2012;159: 477–486. 10.1016/j.trsl.2011.12.003 22633099

[11] Baur JA, Pearson KJ, Price NL, Jamieson HA, Lerin C, Kalra A, et al Resveratrol improves health and survival of mice on a high-calorie diet. Nature. 2006;444: 337–342. 17086191 10.1038/nature05354 PMC4990206

[12] Baur JA, Sinclair DA. Therapeutic potential of resveratrol: the in vivo evidence. Nat Rev Drug Discov. 2006;5: 493–506. 16732220 10.1038/nrd2060

[13] Hung LM, Chen JK, Huang SS, Lee RS, Su MJ. Cardioprotective effect of resveratrol, a natural antioxidant derived from grapes. Cardiovasc Res. 2000;47: 549–555. 10963727 10.1016/s0008-6363(00)00102-4

[14] Das S, Das DK. Resveratrol: a therapeutic promise for cardiovascular diseases. Recent Pat Cardiovasc Drug Discov. 2007;2: 133–138. 18221111 10.2174/157489007780832560

[15] Das S, Das DK. Anti-inflammatory responses of resveratrol. Inflamm Allergy Drug Targets. 2007;6: 168–173. 17897053 10.2174/187152807781696464

[16] Schmatz R, Perreira LB, Stefanello N, Mazzanti C, Spanevello R, Gutierres J, et al Effects of resveratrol on biomarkers of oxidative stress and on the activity of delta aminolevulinic acid dehydratase in liver and kidney of streptozotocin-induced diabetic rats. Biochimie. 2012;94: 374–383. 10.1016/j.biochi.2011.08.005 21864646

[17] Lagouge M, Argmann C, Gerhart-Hines Z, Meziane H, Lerin C, Daussin F, et al Resveratrol improves mitochondrial function and protects against metabolic disease by activating SIRT1 and PGC-1alpha. Cell. 2006;127: 1109–1122. 17112576 10.1016/j.cell.2006.11.013

[18] Um JH, Park SJ, Kang H, Yang S, Foretz M, McBurney MW, et al AMP-activated protein kinase-deficient mice are resistant to the metabolic effects of resveratrol. Diabetes. 2010;59: 554–563. 10.2337/db09-0482 19934007 PMC2828647

[19] Tennen RI, Michishita-Kioi E, Chua KF. Finding a target for resveratrol. Cell. 2012;148: 387–389. 10.1016/j.cell.2012.01.032 22304906

[20] Andrade JM, Paraiso AF, de Oliveira MV, Martins AM, Neto JF, Guimaraes AL, et al Resveratrol attenuates hepatic steatosis in high-fat fed mice by decreasing lipogenesis and inflammation. Nutrition. 2014;30: 915–919. 10.1016/j.nut.2013.11.016 24985011

[21] Chachay VS, Macdonald GA, Martin JH, Whitehead JP, O'Moore-Sullivan TM, Lee P, et al Resveratrol does not benefit patients with nonalcoholic fatty liver disease. Clin Gastroenterol Hepatol. 2014;12: 2092–2103 e1–6. 10.1016/j.cgh.2014.02.024 24582567

[22] Heebøll S, Kreuzfeldt M, Hamilton-Dutoit S, Kjær Poulsen M, Stødkilde-Jørgensen H, Møller HJ, et al Placebo-controlled, randomised clinical trial: high-dose resveratrol treatment for non-alcoholic fatty liver disease. Scand J Gastroenterol. 2016;51: 456–464. 10.3109/00365521.2015.1107620 26784973

[23] Liberati A, Altman DG, Tetzlaff J, Mulrow C, Gotzsche PC, Ioannidis JP, et al The PRISMA statement for reporting systematic reviews and meta-analyses of studies that evaluate health care interventions: explanation and elaboration. PLoS Med. 2009;6: e1000100 10.1371/journal.pmed.1000100 19621070 PMC2707010

[24] Mantel N, Haenszel W. Statistical aspects of the analysis of data from retrospective studies of disease. J Natl Cancer Inst. 1959;22: 719–748. 13655060

[25] DerSimonian R, Laird N. Meta-analysis in clinical trials. Control Clin Trials. 1986;7: 177–188. 3802833 10.1016/0197-2456(86)90046-2

[26] Faghihzadeh F, Adibi P, Hekmatdoost A. The effects of resveratrol supplementation on cardiovascular risk factors in patients with non-alcoholic fatty liver disease: a randomised, double-blind, placebo-controlled study. Br J Nutr. 2015;114: 796–803. 10.1017/S0007114515002433 26234526

[27] Faghihzadeh F, Adibi P, Rafiei R, Hekmatdoost A. Resveratrol supplementation improves inflammatory biomarkers in patients with nonalcoholic fatty liver disease. Nutr Res. 2014;34: 837–843. 10.1016/j.nutres.2014.09.005 25311610

[28] Popov VB, Lim JK. Treatment of nonalcoholic fatty liver disease: the role of medical, surgical, and endoscopic weight loss. J Clin Transl Hepatol. 2015;3: 230–238. 10.14218/JCTH.2015.00019 26623270 PMC4663205

[29] Qiu LX, Chen T. Novel insights into the mechanisms whereby isoflavones protect against fatty liver disease. World J Gastroenterol. 2015;21: 1099–1107. 10.3748/wjg.v21.i4.1099 25632182 PMC4306153

[30] Wang ZQ, Zhang XH, Yu Y, Tipton RC, Raskin I, Ribnicky D, et al Artemisia scoparia extract attenuates non-alcoholic fatty liver disease in diet-induced obesity mice by enhancing hepatic insulin and AMPK signaling independently of FGF21 pathway. Metabolism. 2013;62: 1239–1249. 10.1016/j.metabol.2013.03.004 23702383 PMC3838888

[31] Li L, Hai J, Li Z, Zhang Y, Peng H, Li K, et al Resveratrol modulates autophagy and NF-κB activity in a murine model for treating non-alcoholic fatty liver disease. Food Chem Toxicol. 2014;63: 166–173. 10.1016/j.fct.2013.08.036 23978414

[32] Bo S, Ciccone G, Castiglione A, Gambino R, De Michieli F, Villois P, et al Anti-inflammatory and antioxidant effects of resveratrol in healthy smokers a randomized, double-blind, placebo-controlled, cross-over trial. Curr Med Chem. 2013;20: 1323–1331. 23298135 10.2174/0929867311320100009

[33] Poulsen MM, Vestergaard PF, Clasen BF, Radko Y, Christensen LP, Stødkilde-Jørgensen H, et al High-dose resveratrol supplementation in obese men: an investigator-initiated, randomized, placebo-controlled clinical trial of substrate metabolism, insulin sensitivity, and body composition. Diabetes. 2013;62: 1186–1195. 10.2337/db12-0975 23193181 PMC3609591

