# Supplementary figures and images for: Efficacy of Resveratrol Supplementation against Non-Alcoholic Fatty Liver Disease: A Meta-Analysis of Placebo-Controlled Clinical Trials
Source: PLoS One. 2016 Aug 25;11(8):e0161792. doi: 10.1371/journal.pone.0161792 (PMC4999224; doi:10.1371/journal.pone.0161792)

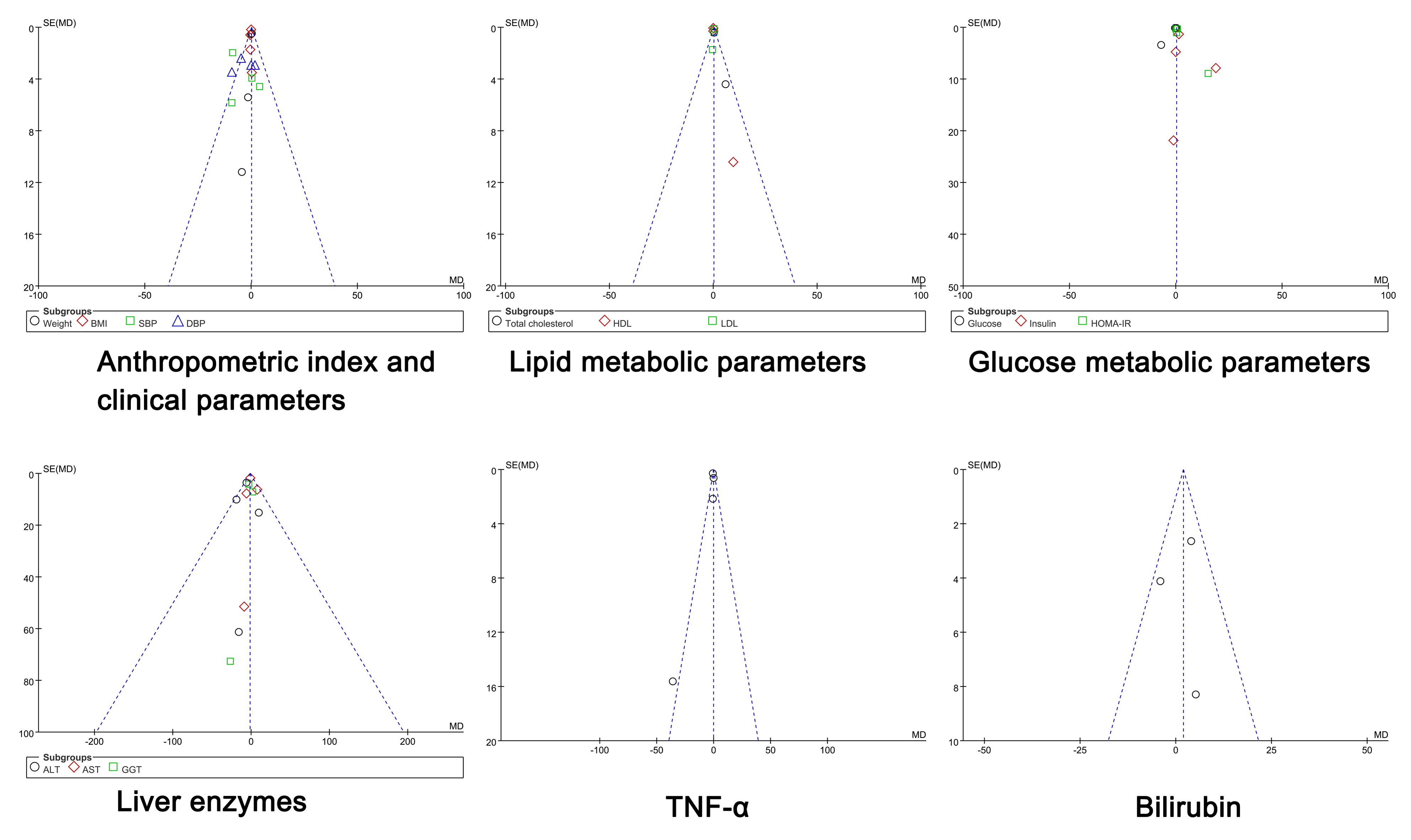

Supplement: S1 Fig — (TIF) [file pone.0161792.s001.tif]

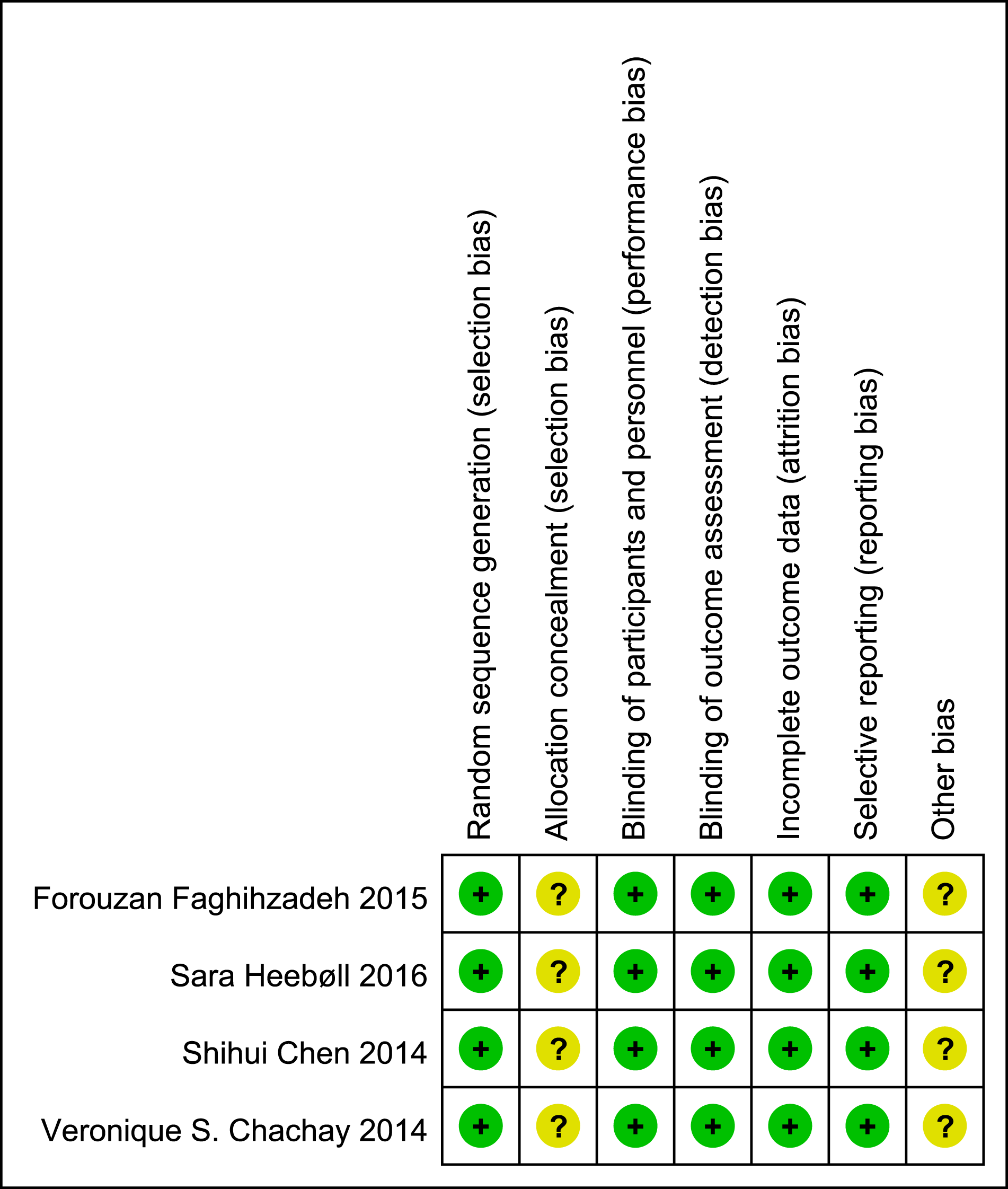

Supplement: S2 Fig — (TIF) [file pone.0161792.s002.tif]

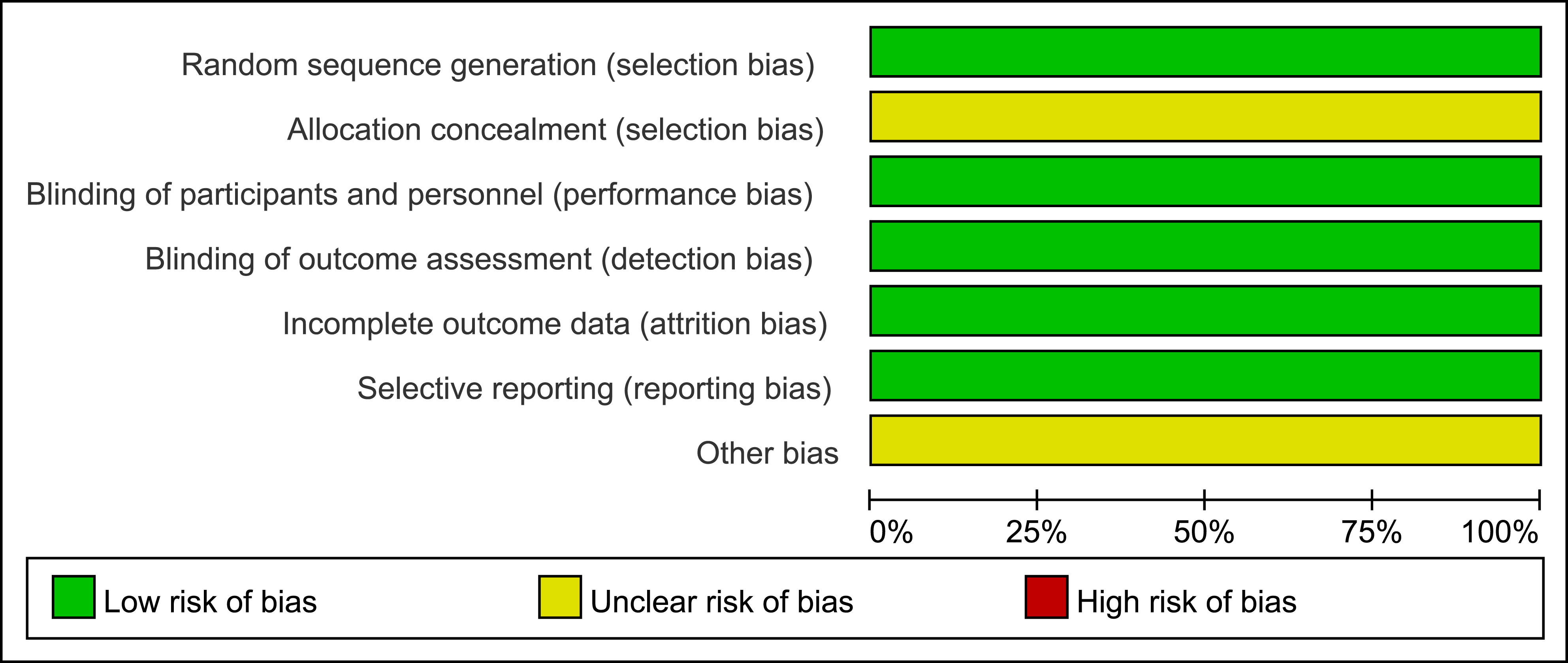

Supplement: S3 Fig — (TIF) [file pone.0161792.s003.tif]
